# Characterization of Extremely Low Frequency Magnetic Fields from Diesel, Gasoline and Hybrid Cars under Controlled Conditions

**DOI:** 10.3390/ijerph120201651

**Published:** 2015-01-30

**Authors:** Ronen Hareuveny, Madhuri Sudan, Malka N. Halgamuge, Yoav Yaffe, Yuval Tzabari, Daniel Namir, Leeka Kheifets

**Affiliations:** 1 Radiation Protection Department, Soreq NRC, Yavne 81800, Israel; E-Mails: ronen@soreq.gov.il (R.H.); yoavyaffe1@gmail.com (Y.Y.); 2 Department of Epidemiology, UCLA School of Public Health, University of California (UCLA), Los Angeles, CA 90024, USA; E-Mail: kheifets@ucla.edu; 3 Department of Electrical and Electronic Engineering, The University of Melbourne, Parkville VIC-3010, Australia; E-Mail: malka.nisha@unimelb.edu.au; 4 Rehovot Center for Gifted Children, Rehovot, Israel; E-Mails: yuvalzabari1999@gmail.com (Y.T.); namir2@zahav.net.il (D.N.)

**Keywords:** EMF, ELF, magnetic fields, cars, transportation, hybrid

## Abstract

This study characterizes extremely low frequency (ELF) magnetic field (MF) levels in 10 car models. Extensive measurements were conducted in three diesel, four gasoline, and three hybrid cars, under similar controlled conditions and negligible background fields. Averaged over all four seats under various driving scenarios the fields were lowest in diesel cars (0.02 μT), higher for gasoline (0.04–0.05 μT) and highest in hybrids (0.06–0.09 μT), but all were in-line with daily exposures from other sources. Hybrid cars had the highest mean and 95th percentile MF levels, and an especially large percentage of measurements above 0.2 μT. These parameters were also higher for moving conditions compared to standing while idling or revving at 2500 RPM and higher still at 80 km/h compared to 40 km/h. Fields in non-hybrid cars were higher at the front seats, while in hybrid cars they were higher at the back seats, particularly the back right seat where 16%–69% of measurements were greater than 0.2 μT. As our results do not include low frequency fields (below 30 Hz) that might be generated by tire rotation, we suggest that net currents flowing through the cars’ metallic chassis may be a possible source of MF. Larger surveys in standardized and well-described settings should be conducted with different types of vehicles and with spectral analysis of fields including lower frequencies due to magnetization of tires.

## 1. Introduction

The transportation system is a possible source of extremely low frequency (ELF) electric and magnetic fields (MF), to which a large proportion of the population is exposed [1,2]. The health effects of MF exposure from transportation systems remain unclear, but there has been some public concern about the MF exposure level from new transportation technologies such as gasoline-electric hybrid automobiles, which are becoming increasingly popular throughout the world. Scientists and policy makers agree that hybrid vehicles are good for the planet. However, some concerns about the potential health risks posed by MF from hybrid cars have been raised [3].

Previous work suggests that major sources of MF in cars include the tires and electric currents [4,5]. The level of MF exposure depends on the position within the vehicle (e.g., proximity to the MF sources) and can vary with different operating conditions, as changes to engine load can induce MFs through changes in electric currents. Scientific investigations of the levels of MF in cars are sparse: only one study evaluated fields only in non-hybrid cars [6], two studies of hybrid cars have been carried out [4,7], and few studies have systematically compared exposures in both hybrid and non-hybrid cars [8,9,10,11,12], some based on a very small number of cars. Many have only been presented at scientific meetings, or in the grey literature, with only two published as peer-reviewed papers.

Vedholm measured the field in seven conventional cars (two of them with the battery underneath the back seat or in the trunk), with engines running idle and the air conditioning turned on [6]. In the left front seat, the magnetic field ranged from 0.05 to 3.9 μT, and in the left back seat it ranged from 0.02 to 3.8 μT. A maximum magnetic field of 14 μT was measured at foot level at the left back seat. The highest values were found in cars with the battery located underneath the back seat or in the trunk.

An Australian study [4] examined the magnetic field in all seats and at the floor level in a hybrid car and found higher fields in the rear compared to the front and higher fields on the left side than on the right side. The maximum magnetic field strength levels were found at a frequency of 12 Hz. A Greek study [7] examined the fields in all four seats and at the feet, chest, and head positions in six hybrid cars, under four driving conditions: stationary and during 20–40, 80–120, and over 120 km/h. The measurements showed that the positions with the higher values were located mainly at the rear seats, at feet level. A remarkable increase in the measured values was observed during braking and speedup in both studies.

Dietrich and Jacobs provide a detailed characterization of a variety of transportation systems, including several conventional and electric cars (mostly prototypes) [8]. Although they report that electric vehicles (cars and light trucks) had average magnetic field levels similar to conventional cars, the majority of tests between the types of vehicles were carried out under different conditions, making comparisons difficult. Further, they report that while low frequency fields are similar and are the dominant component in both types of vehicles, higher frequency EMF levels are markedly higher in the electric vehicles. Austrian scientists evaluated two electric, three hybrid, and two conventional vehicles [9]. For each vehicle, 12 measurements were taken at each of the front seats and one of the rear seats. The measurements were carried out under defined stationary speed/load conditions on a car test bench, during acceleration and braking, and while driving in real traffic, including city, motorway, uphill, and downhill. The authors evaluated the spatial average of magnetic exposure inside the cars and report that MF exposure depends on the arrangement of the electric components and cabling between the battery and engine. In the US, the consumer-product testing organization, Consumer Reports, tested five hybrid and eight non-hybrid cars. Measurements were taken at the driver’s right foot, knee, waist, and head while idling, driving in a simulated stop-and-go city-driving course, and accelerating from 0 to 60 miles per hour. For all vehicles, measurements were highest in the driver’s foot well and second-highest at the waist. Typically, peak readings were highest during braking [10]. Scientists from the Japan Electrical Safety & Environment Laboratories [11] evaluated fields from an electric vehicle, a hybrid vehicle, and a gasoline-electric vehicle. They took measurements in 18 positions within each of the 3 cars while the driving speed was held at a constant 0, 10, 40, or 80 km/h. They report that all cars displayed multiple peak fields, with the first two cars having significant peaks at 5.81 Hz, and the third at 6.29 Hz. A US study evaluated six gasoline and eight electric cars (six of which were hybrids). Each vehicle was fitted with six EMDEX Lite broadband meters, with a 4-s sampling interval. The vehicle was then driven around a rectangular 16.3-km loop with a change in elevation of 105 meters that included both city streets and a high-speed freeway. Overall, fields were higher in electric compared to gasoline cars [12].

The European Health Risk Assessment Network on Electromagnetic Fields Exposure (EFHRAN) recently produced a report on the level of exposure in the European Union [13] that points to a paucity of studies on exposure from transportation. Particularly for hybrid cars, they indicate that data exist only on outdated technologies and should be updated.

The reason for possible differences in MF levels between cars with gasoline, diesel, and hybrid engines needs to be investigated, and this paper addresses this need. ORCHID (Hebrew abbreviation of “National Survey of Magnetic Fields in Israel”) is a project aimed at collecting extensive and reliable information regarding the exposure of children in Israel to power-frequency MFs. The innovation of ORCHID is its educational aspect, which integrates gifted 6th to 10th grade students as active scientific participants in the project. The involvement of children also includes personal research projects designed and implemented by them. This study was designed and carried out in collaboration with two of those children and characterizes the ELF MF levels in diesel, gasoline and hybrid cars and their dependence on different parameters.

## 2. Materials and Methods

MF levels were measured in 11 different car models from eight different car manufacturers: four gasoline (G1, G2, G3, G4), three diesel (D1, D2, D3), and four hybrid (H1, H2, H3, H4). MF levels from one of the hybrid cars (H4) were measured under dissimilar conditions, and thus, data from this car were excluded from analysis, leaving 10 cars with extensive measurements. All measurements were taken under similar conditions with EMDEX-II ELF MF meters that were purchased from Enertech and calibrated by Soreq Nuclear Research Center certified laboratories. Measurements were taken with the meters in broadband mode (40–800 Hz) with a sampling rate of 1.5 s. Each car underwent two types of measurements, “spot measurements” and “continuous measurements.” The cars’ lights were on and the air-conditioning and radio were turned off during all measurements.

### 2.1. Spot Measurements

Spot measurements were conducted mainly to identify and describe sources of MF. Measurements were taken while the car was “standing” (not moving), with the engine turned on and idling in a negligible background field (<0.01 μT). The spot measurements were taken at six different positions: in contact with the engine hood (hood closed), at each of the four seats, and inside the trunk. At each of the six locations, the meter was moved slowly along the whole area or volume, from the floor to the ceiling, to identify both typical and maximal MF levels as well as the location of the highest fields.

### 2.2. Continuous Measurements

Continuous measurements were taken under two “standing” and two “driving” operating conditions. During each of the four conditions, measurements were taken simultaneously by the driver and three passengers sitting in the four seats inside the car. Each of the four individuals had an EMDEX-II meter hanging in a pouch from his neck and positioned near the torso. In the standing mode the two conditions were idling (the accelerator pedal left uncompressed with the engine idling) and 2500 revolutions per minute (RPM) (the accelerator pedal compressed to 2500 RPM of the engine crankshaft). In hybrid vehicles turning the key to the “on” position did not cause the engine to start running in the same manner as it does in non-hybrid vehicles. The idling condition in hybrid vehicles is therefore not well-defined and the 2500 RPM condition does not exist. Each set of measurements was taken for 60 s in a negligible background field (same location as in the spot measurements).

The MF levels for the driving conditions were measured while driving on the same segment of road, approximately 1.3 km long, at two different speeds, namely, 40 km/h and 80 km/h. The particular road segment was chosen for a few reasons. First, despite a nearby high-voltage power line (22 kV) the MFs along the road were very low (around 0.01 μT), probably due to low currents. Moreover, this segment is straight with little automobile traffic. These advantages enabled us to conduct the measurements under stable and well-defined conditions, and to minimize the influence of external disturbances. The driver was asked to maintain the speed as constant as possible. The MF meters began recording the field once the vehicle reached the desired speed (either 40 or 80 km/h), and stopped recording before the vehicle began decelerating. Therefore, moving measurements were taken under approximately constant speeds. To check for repeatability, two sets of measurements were taken at each speed.

For each diesel and gasoline car, a total of six continuous measurement runs (one idling, one at 2500 RPM, two at 40 km/h, and two at 80 km/h) were conducted. A total of five runs were conducted for each hybrid car, because the 2500 RPM condition did not exist. For each car, we synchronized the observations within each run across the four seats based on the timestamps for each recording. This was done by removing a few observations at the beginning and/or at the end of data for certain seats in some runs where measurements did not start and end at the exact same times across all four seats (368 of the 11,503 observations were removed).

The background field for the moving condition was characterized by walking with an EMDEX-II meter and recording the MF level along the same segment of road on which the cars were driven. As MF levels were not assessed on the same day for all cars, the background fields were measured twice on each day that car measurements took place (ranging on average from 0.005 to 0.0165 μT). The average background field was subtracted from each car’s MF driving measurements, and any resulting values less than or equal to zero were coded as 0.001 μT (about 0.6% of measurements). To identify possible variations in measurements due to MF from other cars, an “event” was marked in the EMDEX-II meter by the passenger in the back left seat when another car passed by in either the same or opposite direction (which occurred only few times).

The distribution of MF measurements was highly right-skewed. For each set of measurements (one seat at one condition), arithmetic and geometric means (AM, GM), arithmetic and geometric standard deviations (SD, GSD), 5th and 95th percentiles, and percentage of measurements greater than or equal to 0.2 μT were calculated. The 0.2 μT was chosen as a cut point as the value that while capturing high exposures occurs with reasonable frequency in cars. MF levels were compared by engine type, seat position, and operating condition. Statistical tests were performed to identify differences in average MF levels between groups. Analyses were performed in SAS version 9.3 (SAS Institute, Inc., Cary, NC, USA).

## 3. Results and Discussion

### 3.1. Spot Measurements

**Figure 1:**
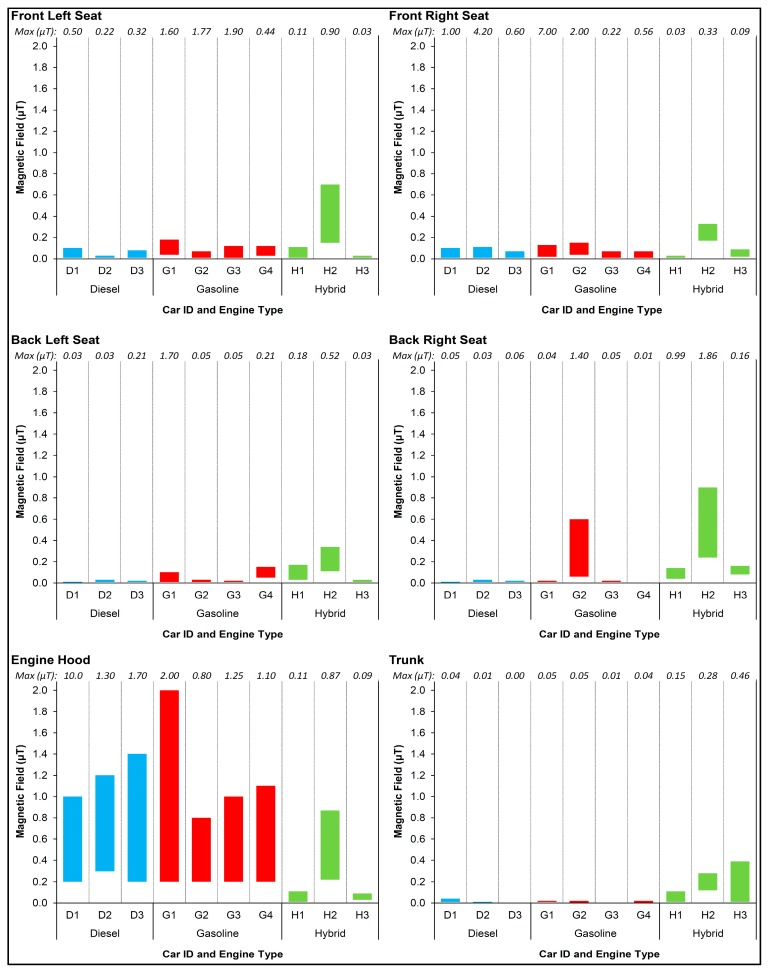
Typical range of MF spot measurements at 6 locations by car and engine type*.*

The following results focus on the upper values of typical range of the spot measurements at each of the six locations in the car. In diesel and gasoline engines, field levels clearly increased when moving from the trunk (mostly in the range of 0.01–0.02 μT), through the back seats (mostly 0.02–0.03 μT) and the front seats (mostly 0.07–0.13 μT), up to the engine hood (typically 1.0–1.4 μT) (Figure 1). In four out of seven cars, the peak fields (of the engine hood) were located near the windshield wiper. Much higher fields were measured closer to the engine itself while the hood was open. The typical spot fields at the four seats were lower for diesel cars than gasoline cars (0.05 and 0.10 μT, respectively).

For hybrid cars, the fields were noticeably higher and less stable relative to the non-hybrids, but we could not find a clear spatial pattern. MFs measured inside the cars were typically 0.05–0.30 μT, and some were even higher (local peaks up to 1.9 μT). Slightly higher fields were found at the back seats (approximately 0.3 μT) than at the front seats (approximately 0.2 μT). Fields at the trunk and near the hood were typically 0.1–0.4 μT.

The maximal fields inside all of the 10 cars were always near the floor (*i.e.*, near the driver’s and passengers’ legs), and in a few cars, local peaks reached 2–10 μT.

### 3.2. Continuous Measurements

After synchronizing readings within each run, a total of 11,140 readings remained for analysis. Results were similar for AM and GM, and given the skewed distributions, we focus on GM and GSD for the remainder of the paper.

As seen in Table 1, the number of observations varies substantially by operating condition (e.g., there are about twice as many observations for 40 km/h as there are for 80 km/h). To evaluate the potential influence of a larger sample on the dispersion around the mean, percentiles, and percent above 0.2 μT, we re-ran some analyses after sampling every-other measurement at 40 km/h. Results were very similar to those obtained from the full dataset (data not shown), and therefore, we present results from the full dataset here.

**Table 1:** Car types and number of continuous measurements by operating condition and seat position.

Model	Engine Type *	Model Year	Number of Measurements							
			By Operating Condition				By Seat Position			
			Idle	2500 RPM	40 km/h ^a^	80 km/h ^a^	Front Left	Front Right	Black Left	Back Right
D1	Diesel	2011	148	148	560	272	282	282	282	282
D2	Diesel 2500	2012	148	148	560	280	284	284	284	284
D3	Diesel 6600	2010	148	148 ^b^	496	272	266	266	266	266
G1	Gasoline 1400	2011	148	148	544	280	280	280	280	280
G2	Gasoline 1600	2010	152	172	592	280	299	299	299	299
G3	Gasoline 1600	2011	148	148 ^c^	528	288	278	278	278	278
G4	Gasoline 1600	2003	148	152	544	280	281	281	281	281
H1	Hybrid 1500	2008	148	0	648	312	277	277	277	277
H2	Hybrid 1340	2009	156	0	576	304	259	259	259	259
H3	Hybrid 1800	2010	156	0	648	312	279	279	279	279

^a^ Repeated measurements combined; ^b^ No RPM meter, pedal was pushed arbitrarily; ^c^ 2500 RPM condition was unstable; * Engine name and engine size in cubic centimeters rounded to nearest 10 (*i.e.*, 1588 rounded to 1600).

**Figure 2:**
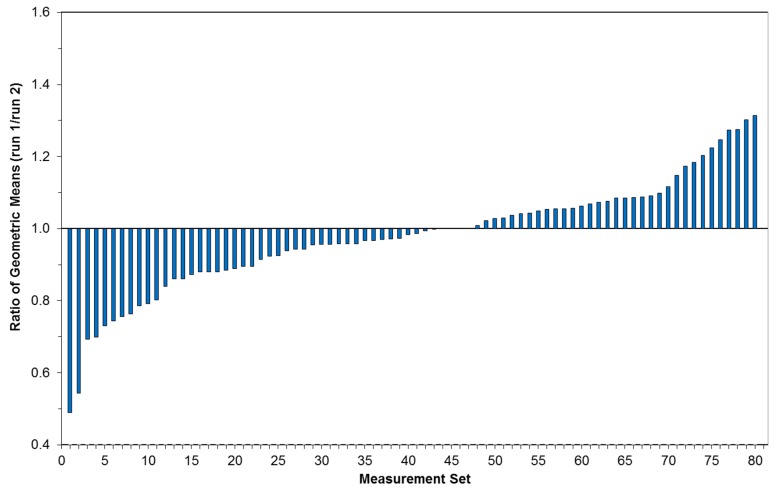
Ratio of geometric means (1st run/2nd run) for 40 km/h and 80 km/h repeated runs, sorted from low to high.

Repeatability of measurements between the first and second moving runs at each speed were evaluated by calculating a ratio of the GM of the measurements from these two runs for each car and seat position (80 sets: four seats, 10 cars, two speeds; Figure 2). We found that measurements were reasonably repeatable, except for the back left and back right seats in car G4 (ratios 0.49 and 0.54) at 40 km/h. *T*-tests comparing runs by car and seat showed that while there were some differences between the runs (as expected given the large dataset and large number of tests), the differences were inconsistent, small and limited to one or two seats in selected runs, thus the first and second runs were combined in the analysis and treated as one larger set for each speed.

Table 2 presents descriptive statistics and *t*-tests for magnetic field measurements overall and by operating condition and engine type. Mean MF levels were significantly higher in hybrid cars. The 95th percentile of MF measurements was also higher in hybrid cars, and the percent of measurements above 0.2 μT was especially high in hybrids. Fields were also significantly lower in diesel cars compared to gasoline cars. For both diesel and gasoline cars, the average MF while standing with the engine revving at 2500 RPM was quite similar to the average while idling. These parameters were higher for moving conditions compared to idling or revving at 2500 RPM, and higher still at 80 km/h compared to 40 km/h regardless of engine type.

**Table 2:** Descriptive statistics for magnetic field measurements (μT) overall and by operating condition and engine type.

	N	Geometric Mean (GSD)	5th Percentile	95th Percentile	% ≥ 0.2 μT
Overall	11,140	0.034 (3.277)	0.006	0.199	4.9
By Engine Type					
Diesel	3328	0.018 (2.912)	0.001	0.059	0.03
Gasoline	4552	0.041 (2.194) ^a^	0.009	0.094	0.29
Hybrid	3260	0.050 (4.385) ^b,c^	0.002	0.427	16.41
By Operating Condition					
Idling	1500	0.028 (3.626)	0.001	0.017	1.33
2500 RPM	1064	0.027 (3.047)	0.001	0.087	0.00
40 km/h	5696	0.030 (3.427) ^d,e^	0.006	0.182	4.07
80 km/h	2880	0.052 (2.565) ^f,g,h^	0.009	0.258	10.31

^a^ Significantly different from Diesel (*t*-test *p <* 0.0001); ^b^ Significantly different from Diesel (*t*-test *p <* 0.0001); ^c^ Significantly different from Gasoline (*t*-test *p <* 0.0001); ^d^ Significantly different from Idling (*t*-test *p =* 0.0831); ^e^ Significantly different from 2500 RPM (*t*-test *p <* 0.0001); ^f^ Significantly different from Idling (*t*-test *p <* 0.0001); ^g^ Significantly different from 2500 RPM (*t*-test *p <* 0.0001); ^h^ Significantly different from 40 km/h (*t*-test *p <* 0.0001).

**Figure 3:**
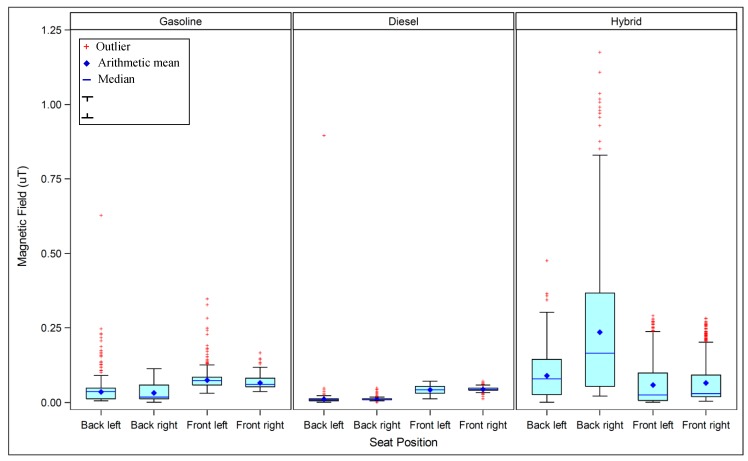
Magnetic field measurement by seat position, stratified by engine type. ^a^ Upper = maximum value less than or equal to 75th percentile + 1.5 * (Interquartile Range); ^b^ Lower = minimum value greater than or equal to 25th percentile 1.5 * (Interquartile Range).

Magnetic field levels in different seat positions varied by engine type. In gasoline and diesel cars, fields were higher in the front seats (Table 3 and Figure 3). Field levels in the driver’s (front left) seats and front passenger seats were very similar to each other in diesel cars, while levels in the driver’s seats were slightly higher than the front passenger seats in gasoline cars (Table 4). For hybrid cars, levels were higher in the back, particularly the back right seat. The difference was striking for the percent of time above 0.2 μT (Table 3). For both gasoline and diesel cars, fields rarely, if ever, reached levels greater than 0.2 μT, regardless of the operating condition or seat position. On the other hand, for hybrid cars, field levels were above 0.2 μT for some amount of time in all seats (except front seats while idling). The percent of time that fields were greater than 0.2 μT in hybrid cars was substantially higher in the back right seat (from 16% to 69%).

**Figure 4:**
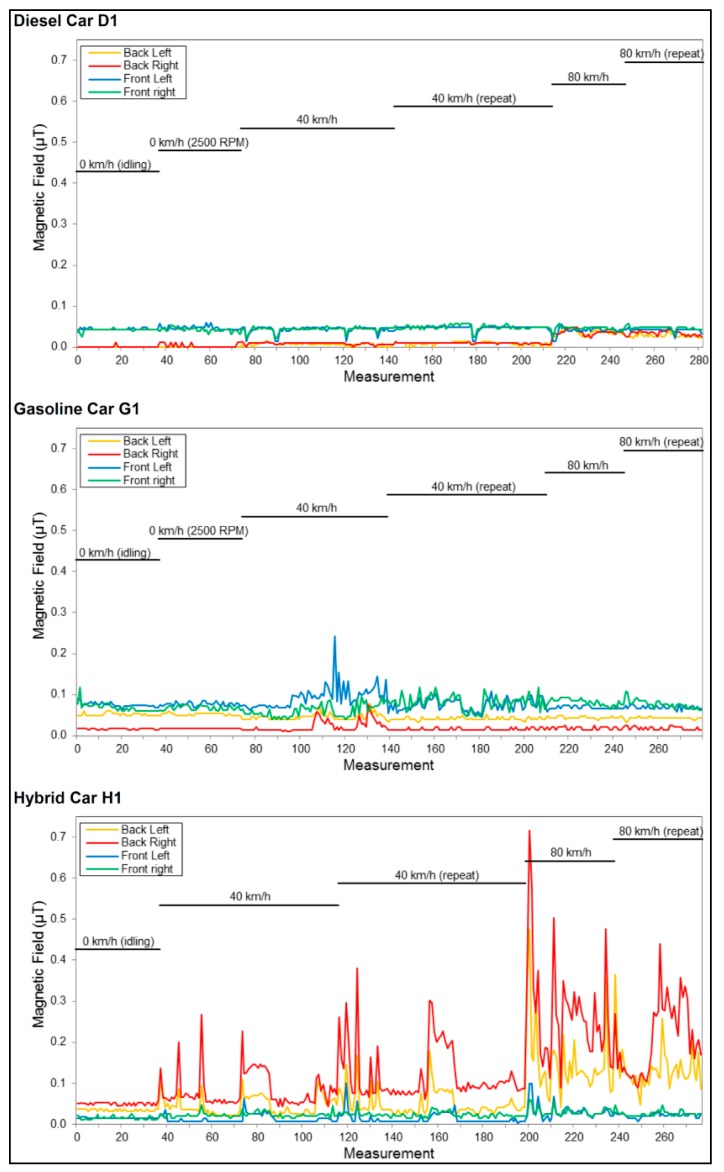
Examples of MF measurements by seat position for three cars; sampling rate of 1.5 s.

Given the difference in exposure by seat, we further evaluated the dependence of magnetic fields on speed. In fact, dependence of magnetic fields on speed varied by engine type (Table 3 and Figure 4). The clearest influence of speed was found for the hybrid cars, in which field levels increased monotonically with speed. Increasing the speed from 40 to 80 km/h increased the fields in all seats in hybrid cars, but particularly in the seat with the highest fields (back right). While the field levels increased with increasing speed for gasoline and diesel cars overall, this trend was not consistent across all seat positions.

To estimate the average MF field levels that might occur during typical driving scenarios, we present five hypothetical scenarios that vary by percent of time spent in each of the three driving conditions–idling, 40 km/h and 80 km/h (Table 5). Scenario A intends to reflect typical highway driving, scenario E reflects typical city driving, and B, C, D are intermediate scenarios. While actual driving conditions may deviate from these examples, we found that overall average MF levels remained similar across all driving conditions, with average fields remaining lowest for diesel cars and highest for hybrid.

**Table 3:** Descriptive statistics for magnetic field measurements (μT) by operating condition and seat position stratified by engine type. Highlighted fields indicate seat position with the highest geometric mean value by engine type and operating condition.

		Diesel			Gasoline			Hybrid		
Operating Condition	Seat Position	Geometric Mean (GSD)	5th–95th Percentile	% > 0.2 μT	Geometric Mean (GSD)	5th–95th Percentile	% > 0.2 μT	Geometric Mean (GSD)	5th–95th Percentile	% > 0.2 μT
Idle	Front Left	0.042 (1.336)	0.026–0.059	0.00	0.073 (1.304)	0.048–0.106	0.00	0.031 (2.678)	0.011–0.136	0.00
	Front Right	0.048 (1.168)	0.038–0.061	0.00	0.062 (1.321)	0.047–0.096	0.00	0.036 (3.100)	0.011–0.182	0.00
	Back left	0.003 (3.965)	0.001–0.018	0.00	0.026 (1.939)	0.011–0.054	0.00	0.040 (3.067)	0.011–0.187	0.87
	Back right	0.005 (3.462)	0.001–0.018	0.00	0.015 (3.069)	0.001–0.064	0.00	0.075 (2.060)	0.037–0.232	16.52
	All seats	0.013 (4.772)	0.001–0.059	0.00	0.037 (2.537)	0.011–0.098	0.00	0.043 (2.875)	0.011–0.198	4.35
2500 RPM	Front Left	0.045 (1.221)	0.036–0.064	0.00	0.073 (1.271)	0.048–0.094	0.00	-	-	-
	Front Right	0.047 (1.195)	0.036–0.061	0.00	0.064 (1.261)	0.047–0.091	0.00	-	-	-
	Back left	0.005 (3.379)	0.001–0.018	0.00	0.023 (1.949)	0.011–0.054	0.00	-	-	-
	Back right	0.007 (3.12)	0.001–0.018	0.00	0.021 (2.094)	0.011–0.064	0.00	-	-	-
	All seats	0.017 (3.737)	0.001–0.061	0.00	0.039 (2.172)	0.011–0.090	0.00	-	-	-
40 km/h	Front Left	0.041 (1.409)	0.021–0.059	0.00	0.073 (1.433)	0.037–0.121	1.27	0.014 (5.815)	0.001–0.195	4.06
	Front Right	0.046 (1.147)	0.037–0.057	0.00	0.064 (1.336)	0.042–0.098	0.00	0.031 (2.773)	0.007–0.175	1.50
	Back left	0.007 (2.009)	0.001–0.013	0.25	0.024 (2.057)	0.009–0.074	0.72	0.029 (5.337)	0.001–0.215	7.69
	Back right	0.009 (1.304)	0.006–0.013	0.00	0.019 (2.382)	0.006–0.071	0.00	0.127 (2.896)	0.030–0.707	33.76
	All seats	0.018 (2.624)	0.006–0.057	0.06	0.038 (2.326)	0.009–0.098	0.50	0.035 (5.116)	0.001–0.413	11.75
80 km/h	Front Left	0.032 (1.829)	0.013–0.064	0.00	0.066 (1.250)	0.044–0.089	0.00	0.058 (2.363)	0.015–0.238	14.66
	Front Right	0.031 (1.684)	0.013–0.049	0.00	0.065 (1.243)	0.044–0.086	0.00	0.067 (2.256)	0.020–0.250	20.26
	Back left	0.016 (1.692)	0.009–0.043	0.00	0.047 (1.435)	0.031–0.071	0.71	0.132 (1.519)	0.075–0.258	22.84
	Back right	0.015 (1.824)	0.009–0.043	0.00	0.037 (1.997)	0.013–0.089	0.00	0.286 (1.706)	0.126–0.688	69.40
	All seats	0.022 (1.944)	0.009–0.054	0.00	0.052 (1.624)	0.021–0.088	0.18	0.11 (2.531)	0.020–0.505	31.79

**Table 4:** Geometric Mean (95% CI) magnetic field (μT) by seat position and engine type; adjusted for car model and operating condition.

Position	Gasoline	Diesel	Hybrid
Black left	0.029 (0.028–0.030)	0.007 (0.007–0.008)	0.055 (0.052–0.058)
Back right	0.023 (0.023–0.023)	0.009 (0.009–0.010)	0.175 (0.166–0.185)
Front left	0.072 (0.070–0.075)	0.039 (0.038–0.041)	0.027 (0.026–0.029)
Front right	0.065 (0.063–0.067)	0.042 (0.041–0.044)	0.046 (0.044–0.049)

**Table 5:** Estimates of Magnetic fields (μT) for typical driving scenarios stratified by engine type.

Scenario	Percent of Time Spent at Each Condition			Geometric Mean (GSD)		
	Idling	40 km/h	80 km/h	Diesel	Gasoline	Hybrid
A	3	22	75	0.021 (2.244)	0.048 (1.834)	0.092 (3.288)
B	5	25	70	0.021 (2.347)	0.048 (1.877)	0.088 (3.383)
C	10	40	50	0.020 (2.631)	0.045 (2.032)	0.073 (3.808)
D	15	55	30	0.019 (2.888)	0.042 (2.176)	0.059 (4.191)
E	18	52	30	0.018 (2.970)	0.042 (2.183)	0.059 (4.126)

## 4. Discussion

In hybrid cars, the battery is generally located in the rear of the car and the engine is located in the front. Electric current flows between these two points through cables that run underneath the passenger cabin of the car. This cable is located on the left for right-hand driving cars and on the right for left-hand driving cars. Although in principle the system uses direct current (DC), current from the alternator that is not fully rectified as well as changes to the engine load, and therefore the current level, can produce MFs which are most likely in the ELF range. While most non-hybrid cars have batteries that are located in the front, batteries in some of them are located in the rear of the car, with cables running to the front of the car for the electrical appliances on the dashboard. In this study, all gasoline and diesel cars had batteries located in the front of the car.

Unlike most ELF exposure sources which generate power frequency (50/60 Hz and harmonic) fields, MFs originating from transportation systems are non-sinusoidal and have a wide spectral range, some at frequencies outside the formal definition of ELF (30–300 Hz), and change rapidly over time. Thus, the exact response curve of the meter used has a crucial influence on the MF results from transportation system surveys. More specifically, the various EMDEX field meters are designed to filter very low frequencies in order to eliminate spurious readings due to movements in the Earth’s static MF. However, as most of the ELF epidemiologic research and surveys have been based on data collected with these meters, it is appropriate to use them to characterize MFs from transportation as well. Thus, our study and some of the other studies of exposures in cars use EMDEX meters [12].

Despite the small number of cars examined, clear exposure differences emerged. Repeated runs resulted in average fields that differ from each other by about 35% or less. As expected, the percent of time above 0.2 µT was the most sensitive parameter of the exposure. Overall, the diesel cars measured in this study had the lowest MF readings (geometric mean less than 0.02 μT), while the hybrid cars had the highest MF readings (geometric mean 0.05 μT). Hybrid cars had also the most unstable results, even after excluding outliers beyond the 5th and 95th percentiles. With regard to seat position, after adjusting for the specific car model, gasoline and diesel cars produced higher average MF readings in the front seats, while hybrid cars produced the highest MF readings in the back right seat (presumably due to the location of the battery). Comparing the different operating conditions, the highest average fields were found at 80 km/h, and the differences between operating conditions were most pronounced in the back right seat in hybrid cars. Whether during typical city or highway driving, we found lowest average fields for diesel cars and highest fields for hybrid cars.

The MFs within cars are quite variable, but their origin and dependence on different parameters are not well understood. To gain a better understanding of some parameters, we held others constant. In particular, our measurements were taken in a well-defined and stable environment, e.g., same position on each seat, low background fields, a single location with little or no traffic, and constant speeds. To accomplish this we avoided accelerations and decelerations. During the measurements we noticed that fields inside the hybrid cars were highly sensitive to any touch of the gas or brake pedals. Hence the fields in hybrid cars seem to be sensitive to both the road and the driver. In real life, accelerating and braking occur frequently, and these will likely increase exposure particularly in hybrid and electric cars [14]. Nevertheless, our estimates of exposure under different driving scenarios are very close to the ones reported by Tell and colleagues (GM [GSD]: 0.051 [2.11] μT) in gasoline cars, and our results for hybrid cars are similar (although a bit lower and more variable) to their measurements in electric cars, most of which were hybrids (GM [GSD]: 0.095 [2.66] μT) [12]. Our results are also consistent with Halgamuge* et al.*, in that they observed higher levels on the left side in right-hand driving hybrid cars [4], while we observed higher fields in the right back seat as the cars in our investigation were the left-hand driving hybrid cars.

Previous works suggest that the magnetization of rotating tires is the primary source of ELF MFs in non-hybrid cars [5,15]. However, the relatively strong fields (on the order of a few μT within the car) originating from the rotating tires are typically at 5–15 Hz frequencies, which are filtered by the EMDEX II meters. Others found that the influence of tire magnetization on the exposure inside the car was negligible [9]. While some contribution of the high harmonic content of the rotating tires to the fields inside the cars is possible, our findings suggest that other sources, possibly the car’s electric current, are the major contributors to the fields. This is true not only for hybrid cars, where the electric power system is an obvious source of the elevated MFs, but also for gasoline and diesel cars. In all the non-hybrid cars, the highest fields from spot measurements were found near the engine hood while idling (typically 1.0–1.4 μT), and the fields decreased monotonically toward the trunk. Additionally, the fields were always maximal near the floor, and in few cases reached 2–10 μT. Highest field levels were also found close to power cables routed near the feet of the occupants in other studies [16]. Based on these observations, we hypothesize that electric currents that are not fully rectified by the alternator might be a major source of MF. Since the car’s electric systems generally use a single cable (from the positive pole), the return current flows through the car’s metallic body, making net currents and stray fields an intrinsic phenomena in cars. Moreover, although the fields that might be generated via this process results only from ripples over the DC currents, the DC currents themselves are high relative to what is used in residential apartments. Due to the low voltage used by cars (usually 12 V), currents can reach many tens of Amperes. This hypothesis was supported by MFs we found over the entire volume of a car standing with its engine turned off, due to a refrigerator installed in the trunk. The unexpected time dependence of the MFs, where most of the peaks are synchronized in all seats (illustrated in Figure 4), also supports this hypothesis.

It should also be noted that there remained some unexplained variability between specific cars with the same engine type. For example, MF levels in one of the hybrid cars (H2) were higher overall. Similarly, there were individual measurements of relatively high fields for some seats at some speeds during particular runs in a few cars. When another car passed by, either in the same or opposite driving direction, one of the passengers marked an “event” on the EMDEX II meter so that the other car’s influence on the measurements could be examined. No noticeable changes were found in the data adjacent to any of the marked events.

Overall, the average MF levels measured in the cars’ seats were in the range of 0.04–0.09 μT (AM) and 0.02–0.05 μT (GM). These fields are well below the ICNIRP [17] guidelines for maximum general public exposure (which range from 200 μT for 40 Hz to 100 μT for 800 Hz), but given the complex environments in the cars, simultaneous exposure to non-sinusoidal fields at multiple frequencies must be carefully taken into account. Nevertheless, exposures in the cars are in the range of every day exposure from other sources. Moreover, given the short amount of time that most adults and children spend in cars (about 30 minutes per day based on a survey of children in Israel (unpublished data), the relative contribution of this source to the ELF exposure of the general public is small. However, these fields are in addition to other exposure sources. Our results might explain trends seen in other daily exposures: slightly higher average fields observed while travelling (GM = 0.096 μT) relative to in bed (GM = 0.052 μT) and home not in bed (GM = 0.080 μT) [1]. Similarly, the survey of children in Israel found higher exposure from transportation (GM = 0.092 µT) compared to mean daily exposures (GM = 0.059 µT). Occupationally, the GM of time-weighted average for motor vehicle drivers is 0.12 μT [18].

As demonstrated by the spot measurements, the results are sensitive to the exact location of the meter, especially to its height. This sensitivity should be considered in the comparison of different studies, as well as in the design of future studies. Our results suggest that further surveys should be conducted with larger samples, in order to verify our results. To obtain valid comparisons, it is important that measurements are performed in standardized and well-described settings. Additional measurements in a variety of other vehicles and electric system configurations (electric cars, busses, trucks, motorcycles,* etc.*) and under various conditions are needed, including during acceleration and deceleration. Special attention should be given to spectral analysis of the fields and to the identification and characterization of the fields’ sources. Further, radio frequency (RF) applications in modern cars are growing and future assessments should include this frequency range as well. Finally, unintentional sources in electric, hybrid and conventional vehicles could be best addressed during vehicle design (e.g., by reducing the battery loop area) [14].

## 5. Conclusions

The results of this characterization of MFs in hybrid and gasoline cars are consistent with previous investigations. For the first time, we report results for diesel cars and characterize the dependence of magnetic field levels on speed. Further, while other studies averaged magnetic field measurements over various seat positions, we describe how fields vary by seat and engine type. In general, MF levels were highest in hybrid cars and lowest in diesel cars. We found that MF levels inside the car’s cabin increased with increasing driving speed and varied by seat position, with the highest levels found in the back seats in hybrid cars and front seats in gasoline and diesel cars. Thus, MF exposure from cars not only depends on the type of engine, but also on operating conditions and the position inside the car.
